# Quality monitoring of intellectual and developmental disabilities systems in the US: Assessing the utility and applicability of selected National Core Indicators to national and state priorities

**DOI:** 10.3389/fresc.2022.960996

**Published:** 2022-08-23

**Authors:** Valerie Bradley, Dorothy Hiersteiner

**Affiliations:** ^1^ President Emerita, Human Services Research Institute, Cambridge, MA, United States; ^2^ Co-Director of National Core Indicators^®^, Human Services Research Institute, Cambridge, MA, United States

**Keywords:** intellectual and developmental disability, disability, performance measurement, quality of life

## Abstract

This article provides historical context on the evolution of performance measures for system improvement, examines the perspectives and insights of state public managers on the use and utility of NQF-endorsed outcome measures from the NCI^®^-IDD In-Person Survey (IPS) for quality improvement, and discusses the necessity of ensuring that outcome measures align with public policy goals.

## Introduction

There is a growing understanding among public administrators of services and supports to people with intellectual and developmental disabilities (IDD) that the best way to assess quality is to ask service participants about their experience. As a result, surveys assessing quality of life and service outcomes based on participant report are increasingly being used to analyze service quality and system performance. The purpose of this article is to explore the use and utility of 14 National Core Indicators®-Intellectual and Developmental Disabilities (NCI®-IDD) measures to the promotion of contemporary public policy goals based on the rankings of state public IDD managers in states across the United States.

**Table 1:** Survey responses.

	Importance to measure and report: Very important or important (*N* = 24)	Usability and use: Very usable or usable (*N* = 24)	Is the measure used or planned to be used for quality improvement in your state?: Yes (*N* = 23)
**NQF domain: Person centered planning and coordination**			
Indicator: The proportion of people who report satisfaction with the level of participation in community inclusion activities	100%	87.5%	82.6%
Indicator: The proportion of people who express they want a job who have a related goal in their service plan	100.0%	83.3%	87.5%
Indicator: The proportion of people who report their service plan includes things that are important to them	91.7%	75.0%	73.9%
Indicator: The proportion of people who express they want to increase independence in functional skills who have a related goal in their service plan	75.0%	75.0%	56.5%
Indicator: The proportion of people who report they are supported to learn new things	75.0%	62.5%	47.8%
**NQF domain: Community inclusion**			
Indicator: The proportion of people who reported that they have friends who are not staff or family members	87.5%	79.2%	60.9%
Indicator: The proportion of people who engage in activities outside the home	87.5%	83.3%	60.9%
Indicator: The proportion of people who reported that they do not feel lonely often	83.3%	62.5%	40.9% (*n* = 22)
**NQF domain: Choice and control**			
Indicator: The proportion of people who report making choices (independently or with help) in life decisions	100.0%	100.0%	65.2%
Indicator: The proportion of people who reported they chose or were aware they could request to change their staff	95.8%	87.5%	60.9%
Indicator: The proportion of people who reported they could change their case manager/service coordinator	95.8%	83.3%	65.2%
Indicator: The proportion of people who live with others who report they can stay home if they choose when others in their house/home go somewhere	79.2%	83.3%	47.8%
**NQF domain: Human and legal rights**			
Indicator: The proportion of people who report that their personal space is respected in the home	100%	100.0%	60.9%

In addition to methodological and psychometric evaluation, reviews of the attributes of outcome measurement systems should also include an analysis of the utility of data collection to inform system improvement. Because policy goals change over time, it is also necessary to periodically review measures to determine their alignment with current policy evaluation needs, research findings, priorities, and aspirations (1).

This article provides historical context for the evolution of performance measurement for system improvement in the United States, examines the perspectives and insights of state public managers on the use and utility of National Quality Forum (NQF)-endorsed outcome measures from the NCI®-IDD In-Person Survey (IPS), and discusses the necessity of ensuring that outcome measures align with public policy goals. While the discussion relies primarily on experiences and practices in the United States, it is hoped that the findings will bolster efforts internationally to ensure that outcome measure remains dynamic and responsive to changing priorities.

### Historical perspective

#### Process standards

To understand the prominence of outcome measurement in the performance of systems of support for people with IDD, it is necessary to reflect on the evolution of quality monitoring in recent decades. As community-based systems expanded in the 1980s and 1990s, public managers developed highly specific process standards intended to embed promising clinical and practice developments into routine practice. This change appeared in state standards for individual habilitation plans, the composition of planning teams, and means to measure progress toward identified goals. Thus, evaluation and monitoring of these standards involved assessing whether certain strategies, steps, processes, and practices were implemented, and did *not* focus on outcomes experienced by the individual or measurement of progress towards individual goals. The concentration on treatment strategies and planning were especially evident in the design of original regulations governing Intermediate Care Facilities for People with Mental Retardation (ICFs/MR).

#### Critique of process standards

As providers became more sophisticated, services and supports less facility-based, and residential arrangements more varied, critics in the field began to chafe under the constraints of rigid and prescriptive process standards. Frustration with ICF/MR standards persisted well into the 1990s (2–6). Researcher Tecla Jaskulski summarized these concerns in a report to the Health Care Finance Administration (7):

• Compliance does not equate with quality
• Standards are not focused on outcomes
• Processes that are reviewed are not sufficiently linked to desired outcomes
• Yes/no dichotomy (i.e., in or out of compliance) ignores individual differences
• Adversarial approach of the survey creates an atmosphere of fault-finding
• There is no focus on continuous quality improvement
• Survey process itself is intrusive in the lives of people with mental retardation

Dissatisfaction with prescriptive process standards led to an exploration of ways to incorporate outcomes into quality assurance assessments. An early examination of the multiple facets of quality monitoring (8) noted that, “Outcome measures are generally seen as the most telling measures to use and as the ultimate basis for legitimizing other approaches to measuring service quality” (p. 17). Authors went on to state, “ … in our analysis of 22 quality assurance systems … few concentrated on outcome measures, and some have not client outcome measures at all” (p. 90).

#### Clinical/functional outcomes

Initial models to better incorporate client or consumer outcomes focused primarily on the extent to which individuals acquired skills or achieved goals, relying on functional scales such as the Scales of Independent Behavior (9) and the Vineland Adaptive Behavior Scales (10). This form of “outcome measurement” was informed by objectives set in individual plans, which in turn reflected expectations of professional providers or caregivers. By the early 1990s, the emphasis again shifted to individual goals such as choice-making, satisfaction, quality of life, and empowerment, moving the locus of assessment from the team to the individual. Rather than focusing primarily on improvement in adaptive skills (or reduction in maladaptive behavior), criteria for the effectiveness of services and supports underscored those outcomes most valued by the individual (11).

### Emergence of quality-of-life outcomes

#### Early initiatives in the United States

A factor that shaped the emerging emphasis of quality-of-life outcomes was the changed expectations and aspirations of people with IDD and their families. These changes could be seen in the advent of self-determination and consumer-directed services in the 1990s. An evaluation of self-determination projects under the Robert Wood Johnson Foundation (12) explored how self-advocates felt about the importance of autonomy in their lives. Self-advocates cited the following key factors enhancing quality of life:

• I am a person like all people: my life is my own.
• I speak for myself. I speak up. I stick up for myself.
• I make my own choices.
• I am the boss of my own life.
• I make my decisions in my own life.
• I do for myself … and not depend on others so much. (p. 4)

These statements reveal that quality of life is a construct that is best understood from the perspective of people receiving services and supports and their family and friends.

An example of this shift from reliance on process and functional outcomes to quality-of-life measures can be seen in the revamped standards published by the Accreditation Council on Services for People with Disabilities (13). Instead of 817 process standards (14) (e.g., advocacy, information and referral, individual records, and plan coordination), the Council proposed a set of ten outcome measures for people and four performance indicators for organizations. Consumer outcomes were (ACDD, 1993, p. 11):

• Personal goals
• Choice
• Social inclusion
• Relationships
• Rights
• Dignity and respect
• Health
• Environment
• Security
• Satisfaction

#### Emergence of National Core Indicators

By the late 1990s, tying performance to outcomes experienced by system participants was widely accepted among advocates, stakeholders, and public managers in the U.S.. What was missing, however, was a common standard tool to canvass participant experiences in a valid and reliable way. Several factors led to the realization of such a tool. First, at the helm of increasingly complex community systems, public managers in IDD sought ways to assess the conduct and impact of supports without actual field observation. Second, improvements in computer capacity made it possible to aggregate and analyze large datasets. Third, state IDD budgets had swelled as the community system expanded, and thus expenditures needed to be justified through results. Finally, an emerging consensus in the field regarding the importance of listening directly to the perspectives and opinions of people receiving supports opened the door for the use of a face-to-face survey administered directly with people receiving services. These factors resulted in the formation of the National Core Indicators® (NCI®) system performance initiative.

In 1997, representatives of 13 state IDD agencies launched an unprecedented effort to create an interstate collaborative for the creation, collection, and analysis of uniform key performance indicators. Since its founding, NCI® has expanded to 48 states, the District of Columbia, and 21 regional developmental disabilities centers in California. NCI® has also grown in its capacity to develop and administer surveys to measure performance of state aging and physical disability systems, measure outcomes experienced by families of people receiving IDD system services and assess the stability and quality of the workforce of direct support professionals. NCI® data make it possible for participating states to track changes in performance, compare outcomes across states, and monitor national trends.

NCI® performance indicators used to measure IDD system performance (heretofore called NCI-IDD) provide a macro-level view of system performance to gauge trends and identify potential gaps. They are intended for use in tandem with other state-administered quality assurance processes under broader quality management systems (e.g., critical incident and reporting systems, risk assessments, provider monitoring, etc.).

To establish the core indicators, state developmental disabilities policymakers identified key criteria to accomplish vital program outcomes. These were designed to: (1) be directly relevant to major organizational or systemic goals; (2) reflect activities that can be influenced by the organization or system; (3) have face validity and should be relevant to the major constituencies served by the organization or system; (4) have directional qualities to reflect changes over time; (5) be expressed as rates or proportions; and (6) include a standard or goal for the desired level of attainment of each outcome (11).

Domains and subdomains within which the indicators are organized reflect major areas of outcomes that affect the mission of public developmental disabilities systems. They include:

Domains and Subdomains:

• Individual outcomes
  o Employment, Community Inclusions and Belonging, Community Participation, Choice and Decision-Making, Relationships, Satisfaction
• System performance
  o Self-Direction, Service Coordination, Workforce, Access
• Health, wellness, and rights
  o Safety, Health, Medication, Rights and Respect
• Family experience
  o Information and Planning, Access and Support Delivery, Workforce, Choice and Decision-Making, Community Connections, Health, Welfare and Safety

As outcome measurement has become an integral component of oversight in public state IDD systems, outcome measures have increasing been accepted as national benchmarks. The Centers for Medicare and Medicaid (CMS) Adult Core Measure Set and the CMS Medicaid and CHIP Scorecard both reflect this important approach (15). The challenge going forward is the extent to which this rich information about participant experience reflects information needed to evaluate the impact of public and in turn is used for both quality improvement and to evaluate the impact of public policy.

### What's next

The previous review of the historical context for the evolution of performance measurement in IDD systems underscores the importance and prominence of quality-of-life measures in conducting oversight of public services and supports for people with IDD. The challenge going forward is to find ways to ensure that measures such as NCI-IDD continue to align with the immediate policy aims of public IDD systems and that they are consistent with the changing context of the provision of services. Lombardi et al. (16) have argued that while the overarching principles that should govern service provision have remained constant over many years, the context within which services operate changes over time as more is learned about best practice and the policies need to achieve those larger system aspirations. Measures therefore need to be reassessed periodically to determine whether they are capturing important contextual elements.

Further, Shogren et al. (17) have described public policy goals as inputs to systems of services and supports, and outcomes as the outputs. According to the authors, this framework (or logic model), allows public managers “to identify core processes that reengineering, quality improvement, and enhanced performance can improve.” Because outcomes or outputs shed light on the efficacy of public policy, it is important to periodically assess whether those results continue to align with public policy goals, as well as whether they are incorporated in the process of quality improvement.

## Method

### NCI^®^-IDD In Person Survey (IPS) measures endorsed by the National Quality Forum

In 1999, the U.S. government created the National Quality Forum (NQF) to advance accountability, patient protection, and quality of care using a variety of measurements and public data reporting. The federal government relies on NQF to review, study, and endorse healthcare-related measures and processes to define government-backed performance and quality measurement strategies. The process for NQF endorsement is rigorous and comprehensive, and measures that achieve endorsement can be relied on to demonstrate strong psychometric properties.

In 2016, NQF released the report, “Quality in Home and Community-Based Services to Support Community Living: Addressing Gaps in Performance Measurement,” calling for increased attention to measures to assess the quality of home and community-based services (HCBS). In the report, NQF defined a measurement framework that included 11 domains and 40 subdomains as areas for quality measurement within HCBS.

In January 2022, NQF approved 14 NCI®-IDD measures following meticulous review of scientific methods, consensus panel analysis, and a public comment period. NQF recognized the high demand for quality measures in home and community-based services, acknowledging the compelling evidence underlying NCI®-IDD measures. The 14 measures are:

**Table:** 

Domain: Person-Centered Planning (PCP) and Coordination
• The proportion of people who express they want a job who have a related goal in their service plan
• The proportion of people who report their service plan includes things that are important to them
• The proportion of people who express they want to increase independence in functional skills (ADLs) who have a related goal in their service plan
• The proportion of people who report they are supported to learn new things
• The proportion of people who report satisfaction with the level of participation in community inclusion activities
Domain: Community Inclusion
• The proportion of people who reported that they do not feel lonely often
• The proportion of people who reported that they have friends who are not staff or family members
• The proportion of people who report adequate transportation
• The proportion of people who engage in activities outside the home
Domain: Choice and Control
• The proportion of people who reported they chose or were aware they could request to change their staff
• The proportion of people who reported they could change their case manager/service coordinator
• The proportion of people who live with others who report they can stay home if they choose when others in their house/home go somewhere
• The proportion of people who report making choices (independently or with help) in life decisions
Domain: Human and Legal Rights
• The proportion of people who report that their personal space is respected in the home

The 14 measures are part of the NCI-IDD In-Person Survey (IPS) which assesses participant outcomes. The survey has three parts. The background section includes sociodemographic, health, employment, and other information that is collected directly from existing administrative records. Section 1 covers more subjective, opinion-based questions that can only be answered by the participant (e.g., Do you like your job? Do you like where you live?). Section 2 contains questions that can be answered by a proxy if the individual is unwilling or unable to respond. This final section relates to more concrete, objective facts, such as the number of times a person went shopping in the community in the past month. In the more than two decades that the survey has been used, approximately two-thirds of respondents have been capable of answering questions without the assistance of a proxy.

The IPS has undergone a number validity and reliability tests and is accompanied by a comprehensive training package. The survey process also includes protocols to detect acquiescent response or “social desirability” including training surveyors to understand whether people are “acquiescing” and if so to rephrase the question in different ways to gather more accurate information. In addition, there is a “proxy determination” section that is designed (and tested) to help surveyors assess whether a proxy is needed (and whether section I should be skipped). This section guides the surveyor to ask non-service-related questions to ascertain the respondent's comprehension and ability to respond accurately.

Each state collects information on and completes a survey with a random sample of individuals that reaches the 5% margin of error and 95% confidence level, based on the total eligible addition population in the state. Individuals are eligible if they receive at least one service from the DD system in in addition to case management. To compare results from state to state, some data are risk-adjusted based on the functional characteristics of individuals served to reduce the impact on aggregate results of differences by state. In addition to recent endorsement by NQF, measures from the IPS have been included in the Medicaid and Children's Health Insurance Program (CHIP) Scorecard and are included in the Medicaid Adult Health Care Quality Measures Core Set (18).

### Survey of public managers

The authors designed a survey to be completed by state public managers to assess their perspectives on the utility and applicability of the NCI®-IDD 14 IPS measures endorsed by NQF. The survey was sent to state DD systems staff who were the state-designated NCI®-IDD liaisons, with instructions suggesting that the initial respondent could consult with other staff in the IDD agency or elsewhere to arrive at the rankings and determine whether the measure was being used for quality improvement.

The survey asked respondents to apply two of the “Criteria for Evaluation” used by NQF (19) for measure endorsement to the 14 endorsed measures. The two criteria were “importance to measure” and “usability and use” and respondents were directed to rank each statement on a five-point Likert scale: 1 = very important, 2 = important, 3 = somewhat important, 4 = not very important, 5 = not important. The survey instructions included definitions of the rating criteria:

• Importance to Measure and Report: Extent to which the specific measure focus is consistent with best practice in the IDD field, is necessary for significant gains in the quality of home and community-based services and improves the quality-of-life outcomes for a specific high-priority aspect of the IDD system where there is variation in or less-than-optimal performance.
• Usability and Use: Extent to which potential audiences (e.g., consumers, purchasers, providers, policymakers) use or could use performance results for accountability and performance improvement to achieve the goal of high-quality, efficient, home and community-based services for people with IDD.

Respondents were then asked, “Is the measure used or planned to be used for quality improvement? If not, why?”

## Results

Twenty-seven (27) responses were received by the close of the survey on April 15, 2022. Of those, two were incomplete, and two were from one state. Respondents to the survey listed a variety of positions and departments, including quality assurance, waiver management, IDD program management, NCI®-IDD liaison, quality improvement, strategic planning, and health planning. State staff that responded to the survey included state home and community-based waiver managers, quality assurance staff, NCI® liaisons, strategic planners, and IDD program consultants.

Table 1 shows results by measure and domain. Measures within domains are ranked based on the proportion of respondents who scored the indicator “very important” or “somewhat important.”

### Important to measure

At least three-fourths of respondents rated all measures as very or somewhat important and four measures were rated very or somewhat important by all respondents. This suggests that public managers believe this subset of measures in the IPS aligns with public policy goals and can be used to assess the performance of services.

### Usability and use

With few exceptions, scores for the usability and use of each measure were likewise fairly high but were lower than the initial rating of importance to measure. Several reasons for this differential may include:

• Current wording of the measure does not adequately reflect the service context
• Results of the measure are not seen as immediately actionable
• Results are not as important to key constituencies

### Measure is used or is planned to be used for quality improvement

While respondents rated the importance of the measures highly, a number indicated that some measures were not currently being used for planning and enhancement, nor were there plans to use the results from that measure in the future. This does not necessarily undermine the measure's potential utility, but rather indicates a need for more intentionality in public systems regarding how to use outcome data to shed light on the achievement of policy goals and to identify elements of service that influence or can influence performance.

If respondents reported that the measure was not being used for quality improvement in their state, respondents were asked to explain why. The following reasons were given:

• New staff were unsure how to use data
• Have not concentrated on that aspect of a particular subdomain
  o “We [use] several questions about level of participation in community inclusion activities, however we do not specifically [use this measure] about satisfaction [with community inclusion].”
• Considering use of the measure in the future
• Has been used inconsistently in the past
  o “[This measure] has been used for QI initiatives but is not consistently used year over year.”
• Measure is of value but not a high priority for strategic planning or quality improvement
• Only so many initiatives that can be managed
• Getting the information from other sources
  o “We ask many questions related to service plans already in a separate QI process, however we do not use this specific [NCI-IDD measure].”
• Do not think the measure is actionable
  o “For [State] this is not an actionable question. It is not clear what the measure would tell us or how we would be able to use the data.”
• Does not reflect what people want
• Do not know how to address, there are other ways of getting at this
• Difficult during COVID (i.e., a measure of the level of engagement in activities out of the home)
• Good for providers and organizations but not for waiver management
  o “[State] HCBS feels that this measure will be useful for providers and organizations in the state, but it won't be useful for HCBS at the state level.”
• Do not use because ratings are high
• Difficult to implement given staff shortage
• Measure is not relevant to state practice
  o “[State] does not focus on this.”
• Could be helpful for settings rule verification

These responses indicate that more general explanations for not actively using particular measures were due to a measure not reflecting state priorities, limited capacity for utilization, and/or COVID-related obstacles to use or relevance. Some respondents also noted that measures were being considered for future analysis, and still others commented on the actionability of the measure, limited avenues for remediating poor results, and lack of alignment with individual goals. It will be important to determine whether these are idiosyncratic problems or serve as a broader critique of the measure and/or its applicability to state quality improvement processes.

## Discussion

### Implications

The results of this preliminary survey suggest that the subset of NCI®-IDD IPS measures endorsed by NQF are seen by public managers as important to measure and, to a slightly lesser extent, are seen to have utility. Fewer respondents, however, note that the measures are actively being used for quality improvement. Obviously, there are only so many measures that can be intentionally tracked and analyzed given limited time and resources. Further, each state's system context may give rise to different priorities. However, some respondents suggested that certain measures do not reflect state practice/policies or there are not realistic ways of remediating negative performance based on the measure.

This survey of state public managers is a first step in a periodic “audit” to determine the viability of outcome measures and their utility to performance and quality measurement. A next step would be an analysis of reasons why some measures were rated lower than others in order to understand the differences in utility, as well as to understand the possible need for more technical assistance to aid public managers in applying survey outcomes to quality enhancement.

### Limitations

This study should be viewed as a qualitative rather than a quantitative examination of the alignment between quality-of-life outcome measures and the current service and policy context. It is meant to start a serious discussion about how to conduct periodic reviews of quality-of-life indicators regarding their useful life and to justify the resources invested in the multi-faceted process of conducting individual interviews. More comprehensive studies of the connections between context and policy on the one hand and outcome indicators on the other will be necessary to avoid measurement for its own sake delinked from public purpose.

## Conclusion

Though Peter Drucker may have never said, “What gets measured gets done” or, alternatively, “What gets measured gets managed,” there is still wisdom in the statement. The outcomes that get measured in the IDD system signal to the field that those outcomes reflect of the values of the system. However, measurement of outcomes should not just signal policy priorities but should be used to evaluate the success of current policies and the need for future policy reform. Outcome measurement should be part of an iterative process that reveals the impact of policies plus the efficacy of elements of the system context – an aspect of the system that Shogren et al. (17) term “influencing factors.” Therefore, it is important for public managers to periodically review whether there is continuity between policy goals and outcomes measurement as well as a collateral review of the processes and practices that are in place to implement those goals. Additionally, it is critical for measure developers to ensure that measures align with policy priorities and are usable.

The challenges highlighted by these results are first, there is a need to work with states to suggest ways to integrate outcome measures into systemic evaluations of state system performance. Secondly, periodic review of outcome measures is important to ensure that they continue to reflect the desired outputs of policy initiatives. Third, negative performance should be accompanied by a more in-depth examination of the system context and the presence or absence of known best practices or “influencing factors.” Shogren et al. (17) use the phrase “outcomes-driven policy” to describe a more comprehensive and robust measurement structure. To ensure the continuing relevance of outcome measurement, it is time to strive for “policy-driven outcomes.”
